# Mucosal nanobody IgA as inhalable and affordable prophylactic and therapeutic treatment against SARS-CoV-2 and emerging variants

**DOI:** 10.3389/fimmu.2022.995412

**Published:** 2022-09-12

**Authors:** Qi Li, Fiachra Humphries, Roxie C. Girardin, Aaron Wallace, Monir Ejemel, Alla Amcheslavsky, Conor T. McMahon, Zachary A. Schiller, Zepei Ma, John Cruz, Alan P. Dupuis, Anne F. Payne, Arooma Maryam, Nese Kurt Yilmaz, Kathleen A. McDonough, Brian G. Pierce, Celia A. Schiffer, Andrew C. Kruse, Mark S. Klempner, Lisa A. Cavacini, Katherine A. Fitzgerald, Yang Wang

**Affiliations:** ^1^MassBiologics, University of Massachusetts Chan Medical School, Boston, MA, United States; ^2^Division of Innate Immunity, Department of Medicine, University of Massachusetts Chan Medical School, Worcester, MA, United States; ^3^Wadsworth Center, New York State Department of Health, Albany, NY, United States; ^4^Department of Biological Chemistry and Molecular Pharmacology, Blavatnik Institute, Harvard Medical School, Boston, MA, United States; ^5^Department of Pathology, University of Massachusetts Chan Medical School, Worcester, MA, United States; ^6^Department of Biochemistry and Molecular Biotechnology, University of Massachusetts Chan Medical School, Worcester, MA, United States; ^7^Institute for Bioscience and Biotechnology Research, University of Maryland, Rockville, MD, United States

**Keywords:** biological sciences, microbiology, SARS-CoV-2, VOC, nanobody, IgA, neutralization, antiviral prophylaxis and therapeutics

## Abstract

Anti-COVID antibody therapeutics have been developed but not widely used due to their high cost and escape of neutralization from the emerging variants. Here, we describe the development of VHH-IgA1.1, a nanobody IgA fusion molecule as an inhalable, affordable and less invasive prophylactic and therapeutic treatment against SARS-CoV-2 Omicron variants. VHH-IgA1.1 recognizes a conserved epitope of SARS-CoV-2 spike protein Receptor Binding Domain (RBD) and potently neutralizes major global SARS-CoV-2 variants of concern (VOC) including the Omicron variant and its sub lineages BA.1.1, BA.2 and BA.2.12.1. VHH-IgA1.1 is also much more potent against Omicron variants as compared to an IgG Fc fusion construct, demonstrating the importance of IgA mediated mucosal protection for Omicron infection. Intranasal administration of VHH-IgA1.1 prior to or after challenge conferred significant protection from severe respiratory disease in K18-ACE2 transgenic mice infected with SARS-CoV-2 VOC. More importantly, for cost-effective production, VHH-IgA1.1 produced in *Pichia pastoris* had comparable potency to mammalian produced antibodies. Our study demonstrates that intranasal administration of affordably produced VHH-IgA fusion protein provides effective mucosal immunity against infection of SARS-CoV-2 including emerging variants.

## Introduction

SARS-CoV-2 is a coronavirus that has led to a global pandemic and causes a severe respiratory disease known as COVID-19. The rapid spread of SARS-CoV-2 globally has resulted in hundreds of millions of infections and over 6.1 million deaths as of March 2022 (1). Despite the rollout of first-generation vaccines and monoclonal antibody therapeutics, additional preventive modalities are still required for breakthrough infections and unvaccinated individuals. Newly emerged SARS-CoV-2 variants of concern (VOC) and interest (VOI) are continuing to evolve globally, including some in which the effectiveness of monoclonal antibodies and vaccines is diminished (2–6). The latest and heavily mutated Omicron and its sub-variants, also exhibit increased transmissibility and risks of infection (7). Thus, new broad variant-resistant treatments and non-invasive delivery strategies remain a high priority (8–10).

Clinical trials have demonstrated that SARS-CoV-2 receptor-binding domain (RBD) targeted neutralizing IgG monoclonal antibodies (MAbs) are safe and effective against COVID-19. Pre- or post-exposure treatment with neutralizing IgG antibodies provide immediate immunity against SARS-CoV-2 in vulnerable patient populations (11, 12). A number of IgG antibodies have received emergency authorization for clinical use (13). However, emerging SARS-CoV-2 VOC continue to diminish the effectiveness of these antibodies (8–10). Intravenous infusion of IgG is not only invasive but also costly with the traditional CHO cell bio-manufacturing platform. Recently, our group demonstrated that compared to IgG, a human IgA monoclonal antibody, MAb362, is more potent at neutralizing SARS-CoV-2 in immunoglobulin’s natural mucosal form: secretory IgA (14). This study raised the possibility of using mucosal IgA as prophylactic therapy against SARS-CoV-2 directly at the infection sites of the respiratory tract. The relevance of this to SARS-CoV-2 infection has been the demonstration that breakthrough infections in vaccinated individuals were seen more frequently in those with lower serum IgA responses to RBD (15). Furthermore, intranasal vaccine boost elicited significantly stronger mucosal IgA responses and provided complete protection of mice from infection (16). Given that SARS-CoV-2 is a respiratory infection, the mucosal response may be more contributory to protection than what is measured in the serum.

The camelid heavy-chain-only antibodies (known as nanobodies or VHHs) are a specific alternative class of monoclonal antibodies, which are single-domain antigen binding fragments derived from Alpaca and Llama. These antigen-binding variable domains are relatively small (~15 kDa), soluble, and highly stable with no associated light chains. Like conventional monoclonal antibodies, nanobodies have emerged as very promising antibody-based therapeutic treatments, diagnostic tools or delivery systems for many diseases, including cancer, infectious disease, neurodegenerative disorders, immune diseases and rare blood diseases (17–27). Compared to monoclonal antibodies, nanobodies are unique biologics that often recognize conserved epitopes on hypervariable pathogens. Due to their smaller paratope diameters and longer complementarity-determining region 3 (CDR3), nanobodies can access structurally distinct, spatially restricted epitopes, such as highly conserved epitopes in recessed regions of viral glycoproteins (28, 29). These unique biophysical advantages have led to the evaluation of mucosal delivery of nanobodies for preventing/treating respiratory pathogens, including respiratory syncytial virus (RSV), whereby nebulized nanobodies greatly reduced RSV infection in newborn lambs (30, 31).

To date, there is no mucosal delivered therapeutic nanobody that has been approved by the FDA. In 2019 Caplacizumab (Sanofi), the first nanobody based drug, was approved by FDA to treat acquired thrombotic thrombocytopenic purpura (aTTP) *via* intravenous and subcutaneous injection (20, 32). Considering more than a dozen nanobodies are currently at different stages of clinical development (18), nanobodies are progressively being demonstrated as a valid clinical alternative to monoclonal antibody therapy to treat many different diseases. Furthermore, nanobodies can be robustly produced in low-cost bio-manufacturing platforms such as yeast or soybean. Mucosal administration of nanobodies produced in yeast has been shown to be safe and effective in reducing severe rotavirus-associated diarrhea in human clinical trials (33), and engineered nanobodies such as nanobody-IgA fusions (VHH-IgA) have proven effective activity for mucosal protection from infection (34). Moreover, antibody production cost in yeast compared to traditional CHO cell may be greatly reduced, resulting in more affordable medicine for pandemic diseases like COVID19 (35–37). Thus, mucosal delivery of engineered nanobodies represents a potentially non-invasive, low cost and effective prophylaxis and post-exposure treatment for respiratory pathogens that could be leveraged for the prevention and therapeutic treatment of SARS-CoV-2 infection.

In this study, we generated a human immunoglobulin constant region fused nanobody using our recently identified affinity matured clone VHH1.1 (38). VHH1.1 monomer was derived from an open source naïve nanobody library (39). The clone was selected based on probing the library with the RBD of the SARS-CoV-2 Spike protein, and further affinity was matured using AHEAD technology as previously described (38). When compared to the original monomeric VHH1.1, the human immunoglobulin Fc region (IgG or IgA) fused nanobody showed significantly increased binding *in vitro* against the spike protein of major SARS-CoV-2 variants. The mutational epitope scanning and receptor blocking results consistently indicated that VHH-IgA1.1 binds to SARS-CoV-2 RBD with high affinity competing with hACE2 receptor. For the *in vitro* neutralization activity, VHH-IgA1.1 exhibited more potent inhibitory activity than the monomeric nanobody and IgG-Fc fusion against SARS-CoV-2 and other VOC, including Alpha, Beta, Gamma and the current dominant circulating Omicron and its new sub variants. Consistent with the *in vitro* results, intranasally administered single dose of VHH-IgA1.1 provided significant protection from infection with SARS-CoV-2 VOC, including Alpha and Omicron variants, in both prophylaxis and treatment mice models. Moreover, to evaluate rapid, large-scale cost-effective manufacturing, we have demonstrated that the yeast *Pichia pastoris* *(P. pastoris)* produced VHH-IgA1.1 has comparable potency against SARS-CoV-2 as conventional mammalian cell produced antibody. Thus, our study provides a potential rapidly scalable, cost-effective mucosal delivered prophylactic and anti-viral treatment for COVID-19. Moreover, our study provides a novel platform for rapid development of biologics against SARS-CoV-2 and other respiratory pathogens in a cost and time effective way.

## Results

### VHH-IgA1.1 fusion binds to spike protein of SARS-CoV-2 and VOC with high affinity

**Figure 1:**
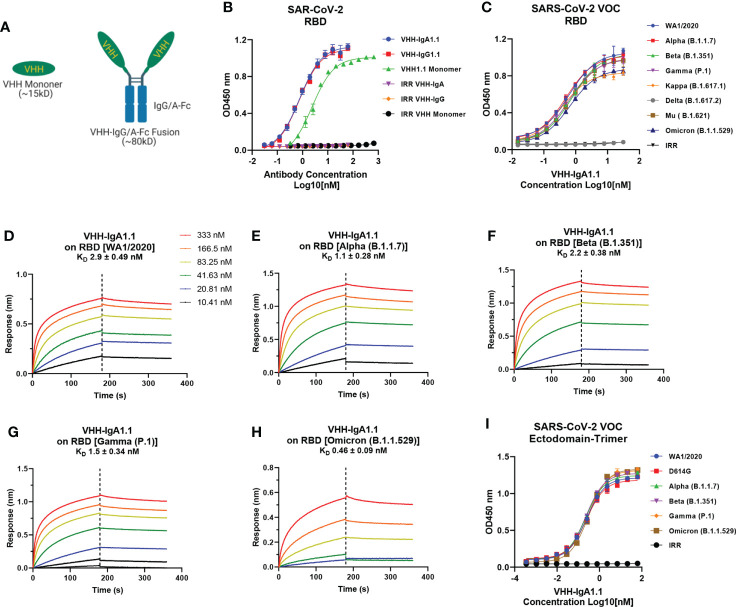
Binding of VHH-IgA1.1 to spike proteins of SARS-CoV-2 and VOC. **(A)** Illustration of VHH engineering from monomer into IgG or IgA-Fc fusions. **(B)** Comparison of VHH1.1 monomer, VHH-IgG1.1 and VHH-IgA1.1 binding with SARS-CoV-2 RBD domain in ELISA. **(C)** ELISA binding of VHH-IgA1.1 with RBD and soluble ectodomain trimer **(I)** generated from indicated SARS-CoV-2 VOC strains. **(D–H)** Affinity measurements of VHH-IgA1.1 against RBD of SARS-CoV-2 VOC were conducted using bio-layer interferometry. Data is plotted as the average ± SD from at least 3 independent experiments.

To explore the potential of VHH1.1 as a prophylactic candidate, we engineered the VHH1.1 monomer sequence onto the Fc region of human IgG1 and IgA1 and expressed the fusion protein in Expi293 cells (**Figure 1A**). Strikingly, the chimeric VHH1.1, both VHH-IgG1.1 and VHH-IgA1.1, showed over 3-folds increased binding activity against RBD antigen of SARS-CoV-2 (VHH-IgG1.1 EC_50 =_ 0.737nM and VHH-IgA1.1 EC_50 =_ 0.774nM) when compared to the monomeric VHH1.1 (EC_50 =_ 2.359nM) **(** **Figure 1B****).** Given enhanced binding potency, VHH1.1 Fc fusions were tested on the cross-binding activity against spike proteins of SARS-CoV-2 VOC, including the RBD of WA1/2020, Alpha (B.1.1.7), Beta (B.1.351), Gamma (P.1), Kappa (B.1.617.1), Delta (B.1.617.2), Mu (B. 1.621) and Omicron (B.1.1.529) strains. Strikingly, IgA form of VHH1.1 bound to all tested RBDs except Delta (**Figure 1C****)** with an affinity range from 0.46 to 2.9nM (**Figures 1D–H**), but VHH-IgG1.1 showed notably decreased binding against Omicron variant (**Figures S1C–E**). Since the purity and homogeneity of purified VHH-IgA1.1 and VHH-IgG1.1 were confirmed by SDS-PAGE and HPLC-SEC (**Figures S1A, B**), this binding activity improvement indicates that VHH1.1 fused with human IgA constant region could potentially benefit the antibody potency, and this isotype related enhanced potency has been reported previously (14, 40). To confirm the RBD binding results, VHH-IgA1.1 were tested against the soluble ectodomain trimers (a.a.1-1208) of SARS-CoV-2 spike protein. Comparable binding activities were observed among SARS-CoV-2 and all tested VOC spike proteins (**Figure 1I**). In addition, VHH-IgA1.1 retained potent hACE2 receptor blockade activity when compared to the monomer (**Figures S2A, B–D**) or IgG-Fc fusion (38). Thus, VHH-IgA1.1 is likely to interact with a conserved epitope within the RBD of SARS-CoV-2 and known variants.

To delineate the binding epitope of VHH-IgA1.1 on SARS-CoV-2 RBD, mutagenesis scanning was performed with a combination of alanine (to introduce a loss of interaction), tryptophan (to introduce a steric challenge), and lysine (to introduce charge) mutations. Residues including D442, L452, Y449, Y453, R466 and Y473 were found to be critical for complex formation, as mutations at these positions caused marked loss of binding affinity (**Figure S3A**). When compared with the hACE2 receptor interacting interface on RBD (**Figures S3B, C**), the key binding residues of VHH-IgA1.1, including Y449, Y453 and Y473 overlapped with the hACE2 interface, and L452 was reported to disrupt the hACE2 binding indirectly (41). Thus, the mutational epitope screening results were consistent with the strong activity of VHH-IgA1.1 compromising the RBD-receptor interaction.

### VHH-IgA1.1 exhibits potent neutralization activity against SARS-CoV-2 VOC *in vitro*

**Figure 2:**
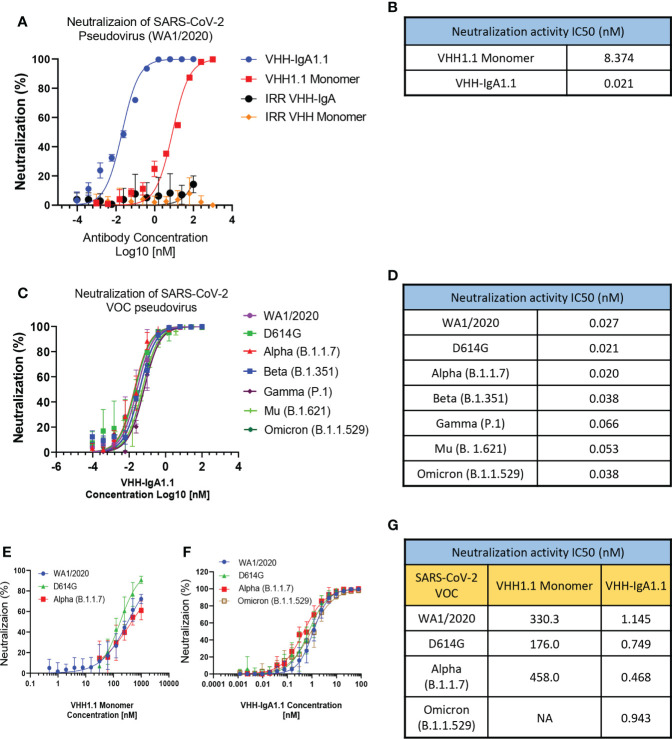
VHH-IgA1.1 potently neutralizes SARS-CoV-2 VOC. Monomeric VHH1.1 and VHH-IgA1.1 mediated *in vitro* neutralization of luciferase-encoding pseudovirions with full length spike proteins presented in SARS-CoV-2 **(A)** and indicated VOC **(C)**. Pseudoviral transduction was measured by luciferase activities to calculate neutralization (%) relative to non-antibody-treated controls. Dose-Response Curve generated from PRNT of monomeric VHH1.1 **(E)** and VHH-IgA1.1 **(F)** against indicated authentic virus of SARS-CoV-2 VOC on Vero E6 cells. **(B, D**, **G)** Data was plotted as the average ± SD, and IC_50_ values were calculated by nonlinear regression analysis. Three or more independent biological replicates were completed for each antibody. NA=not tested.

Given the broader and stronger binding activity of VHH-IgA1.1 against major SARS-CoV-2 VOC than the IgG equivalent, we next evaluated VHH-IgA1.1 mediated neutralization of SARS-CoV-2 variants in a lentiviral based pseudovirus assay on hACE2 transfected 293T cells. The efficacy of VHH-IgA1.1 was expressed as the concentration capable of inhibiting 50% of pseudovirus entry (IC_50_). Compared to an irrelevant VHH-IgA control, VHH-IgA1.1 showed potent neutralization activity with an IC_50_ of 0.021nM, which is about 400 times more potent than monomeric VHH1.1 (**Figures 2A, B**). More importantly, VHH-IgA1.1 retained strong neutralization activity against pseudovirus expressing spike proteins of SARS-CoV-2 VOC, including D614G, Alpha, Beta, Gamma, Mu and Omicron with an IC_50_ ranging from 0.020 to 0.066nM (**Figures 2C, D**). Compared with IgA form, VHH-IgG1.1 exhibited relatively weaker neutralization activity against pseudovirus of SARS-CoV-2 variants (**Figures S1F–L**). Notably, consistent with binding results (**Figure S1E**), the neutralization potency of VHH-IgG1.1 was 17 times less than VHH-IgA1.1 against the Omicron variant (**Figure S1L**). This result suggested that VHH fused with IgA Fc could also substantially enhance the neutralization potency.

The neutralization efficacy of monomeric VHH1.1 and VHH-IgA1.1 against SARS-CoV-2 authentic virus were tested by Plaque Reduction Neutralization Tests (PRNT). Live, authentic virus isolates from WA1/2020, D614G, Alpha and Omicron lineages were used to infect Vero E6 cells in the presence of antibodies. Both VHH1.1 monomer and VHH-IgA1.1 treated Vero cells had a significant reduction in plaques (**Figures 2E, F**). VHH-IgA1.1 had an IC_50_ ranging from 0.468 to 1.145nM, which was 234-978 times more potent than monovalent VHH1.1 (**Figure 2G**), and this potency improvement is consistent with the pseudovirus neutralization results (**Figure 2B**). For the newly emerged Omicron sublineages, such as BA.1.1, BA.2 and BA.2.12.1, the efficacy of VHH-IgA1.1 was maintained. The neutralization IC_50_ of VHH-IgA1.1 against those 3 Omicron sublineages were still at nanomolar range (<10nM) in PRNT assay. Thus, VHH-IgA1.1 potently neutralized both pseudotyped and authentic SARS-CoV-2, including major known VOC *in vitro.*

### Intranasal administration of VHH-IgA1.1 protects animals from SARS-CoV-2 variants infection

**Figure 3:**
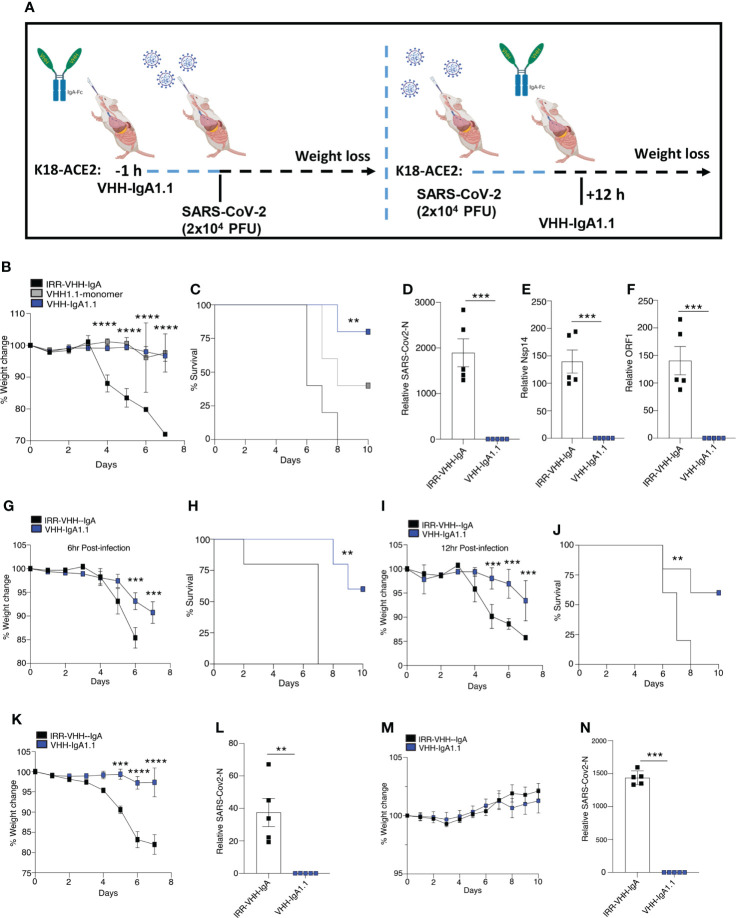
Single-dose intranasal VHH-IgA1.1 protects K18-ACE2 transgenic mice from SARS-CoV-2 infection. **(A)** Schematic of VHH-IgA1.1 intranasal delivery and SARS-CoV-2 infection. (**B, C)** Weight loss **(B)** and survival **(C)** of K18-ACE2 transgenic mice infected intranasally with SARS-CoV-2 (2.5x10^4^ PFU/mouse) with a 1 h intranasal pre-treatment of 10mg/kg IRR-IgA isotype control (*n=5*), VHH-monomer (*n=5*) or VHH-IgA1.1 **(D-F)** QPCR analysis of SARS-CoV-2-N **(D)**, Nsp14 **(E)** ORF1 **(F)** in lung tissue of K18-ACE2 transgenic mice infected with SARS-CoV-2 for 48 h with a 1 h intranasal pre-treatment of 10mg/kg IRR-IgA1 isotype control (*n=5*) or VHH-IgA1.1 (*n=5*). **(G–J)** Weight loss and survival of K18-ACE2 transgenic mice infected intranasally with SARS-CoV-2 (2.5x10^4^ PFU/mouse) and treated with 10mg/kg IRR-IgA1 isotype control (*n=5*) or VHH-IgA1.1 (*n=5*) 6 h **(G, H)** or 12 h **(I, J)** after infection. (**K, M**) Weight loss of K18-ACE2 transgenic mice infected intranasally with Alpha **(K)** and Omicron **(N)** (1x10^5^ PFU/mouse) variant of SARS-CoV-2 followed by pre-treatment of 10mg/kg IRR-IgA1 isotype control (*n=5*) or VHH-IgA1.1 (*n=5*). **(L**, **N)** QPCR analysis of SARS-CoV-2-N in lung tissue of K18-ACE2 transgenic mice infected with SARS-CoV-2 Alpha **(L)** and Omicron **(N)** for 48 h with a 1 h intranasal pre-treatment of 10mg/kg 10mg/kg IRR-IgA1 isotype control (*n=5*) or VHH-IgA1.1 (*n=5*). **p<0.001 ***p<0.0001, ****p<0.00001. (**C**, **H**, **J** Mantel–Cox survival analysis). Error bars show means ± SEM.

Given that VHH-IgA1.1 neutralized SARS-CoV-2 *in vitro* more potently than the monomeric and IgG-Fc fused nanobody, we next determined if VHH-IgA1.1 could also neutralize SARS-CoV-2 *in vivo* and protect against severe disease. Since SARS-CoV-2 cannot infect cells *via* murine ACE2, SARS-CoV-2 infection cannot be established in regular laboratory mouse strains (42, 43). K18-hACE2 transgenic mice (K18-hACE2), in which human ACE2 is expressed under the control of the epithelial cell cytokeratin-18 (K18) promoter (44) were used to establish an *in vivo* prophylaxis and post-exposure treatment model (**Figure 3A**). As previously reported (45, 46), K18-ACE2 Tg mice inoculated intranasally with SARS-CoV-2 (WA1/2020) resulted in rapid weight loss and lethality 8 days post-infection (**Figures 3B, C**). However, K18-ACE2 Tg mice pre-treated with a single intranasal dose of VHH1.1 monomer or VHH-IgA1.1 at 10 mg/kg were protected from SARS-CoV-2 induced weight loss and lethality when compared to isotype control (**Figures 3B, C**). In line with our *in vitro* data, VHH-IgA1.1 was more effective than monomeric VHH1.1. Remarkably, VHH-IgA1.1 also protected mice from SARS-CoV-2 induced weight loss and lethality when administered 6 h (**Figures 3G, H**) or 12 h post-infection (**Figures 3I, J**) as treatment. A dose range analysis of VHH-IgA1.1 also demonstrated potent protective effects with protection from weight loss and lethality retained at 5mg/kg, 2.5mg/kg and 1mg/kg (**Figures S4A–B**). Given these results, we next assessed the effect of VHH-IgA1.1 on viral loads in lung tissue. Single intranasal pre-treatment of K18-ACE2 mice with VHH-IgA1.1 one hour prior to challenge resulted in a decrease in the expression of SARS-CoV-2 genes including N-protein (**Figure 3D**), Nsp14 (**Figure 3E**) and ORF1 (**Figure 3F**) in lung tissue collected 48 h post-infection. Next, we assessed the neutralization efficacy of VHH-IgA1.1 against two SARS-CoV-2 VOC, Alpha and Omicron *in vivo*. VHH-IgA1.1 protected mice from SARS-CoV-2 Alpha induced weight loss (**Figure 3K**) and lethality (**Figure S5A**) when compared to the isotype control. VHH-IgA1.1 treatment also resulted in a significant reduction of viral replication in lung tissue 48h post-infection (**Figure 3L**). Omicron has been identified as a less virulent variant of SARS-CoV-2, and infection with Omicron causes attenuated disease in K-18 ACE2 transgenic mice and hamsters (47). Indeed, in line with previous studies mice infected with Omicron displayed no weight loss (**Figure 3M**) or lethality (**Figure S5B**). However, despite attenuated disease Omicron was detected in lung tissue 48h post-infection (**Figure 3N**). In addition, Omicron viral RNA was significantly reduced in mice pre-treated with a single intranasal dose of VHH-IgA1.1 (**Figure 3N**). Thus, VHH-IgA1.1 delivered prophylactically or therapeutically exhibited potent neutralization activity against SARS-CoV-2 and VOC *in vivo.*

### *P. pastoris* produced VHH-IgA1.1 potently neutralizes SARS-CoV-2

**Figure 4:**
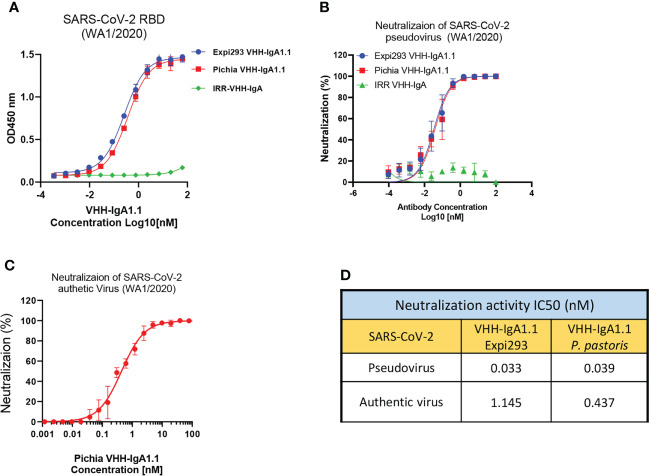
*P. pastoris* produced VHH-IgA1.1 exhibits potent activity against SARS-CoV-2. **(A)** Comparison of Expi293 cells and *P. pastoris* produced VHH-IgA1.1 binding to SARS-CoV-2 RBD in ELISA. **(B)** Comparison of Expi293 cells and *P. pastoris* produced VHH-IgA1.1 mediated *in vitro* neutralization of luciferase-encoding pseudovirions with full length spike proteins presented in SARS-CoV-2 WA1/2020 strain. **(C)** Dose-Response Curve generated from PRNT of *P. pastoris* produced VHH-IgA1.1 against authentic SARS-CoV-2 on Vero E6 cells. **(D)** Data was plotted as the average ± SD, and IC_50_ values were calculated by nonlinear regression analysis. Three or more independent biological replicates were completed for each antibody.

Given its rapid, large-scale, and cost-effective production methods, production of full length human antibodies and nanobodies in methylotrophic yeast *P. pastoris* has become an increasingly popular expression system over traditional production methods (48). Thus, we engineered the VHH-IgA1.1 into a pPINKa-HC system and expressed it in *P. pastoris*. The binding activity of *P. pastoris* and mammalian cell produced VHH-IgA1.1 against SARS-CoV-2 RBD were tested head-to-head, and remarkably, both antibodies exhibited comparable binding activity (**Figure 4A**). Consistently, both *P. pastoris* and mammalian cell produced VHH IgA-Fc fusion antibody neutralized pseudotyped and authentic SARS-CoV-2 WA1/2020 strain comparably *in vitro* (**Figures 4B, C**). Notably, the *P. pastoris* VHH-IgA1.1 had over 2-fold enhanced potency against authentic SARS-CoV-2 live virus when compared to mammalian cell derived VHH-IgA1.1 (**Figure 4D**). Thus, our results demonstrate that the production of functional recombinant IgA-Fc fusion nanobodies can be achieved using the rapid, scalable, and cost-effective *P. pastoris* expression system.

## Discussion

Collectively, our data demonstrates that intranasal delivery of a neutralizing nanobody IgA-Fc fusion protects animals from infection of SARS-CoV-2 VOC, and the results strongly suggest that the IgA-Fc fused nanobody provides a greater level of protection in the mucosal lining of the nasal passages and lungs, as either an immunoprophylaxis or post-exposure treatment, than the monomeric nanobody. This result is consistent with most recent nanobody studies against SARS-CoV-2, which have shown that multimerization or Fc fusion could significantly increase the efficacy of therapeutic nanobodies (49–60). Since the spike proteins are naturally presented as a trimer on the surface of SARS-CoV-2, one possibility is that the multimerized or Fc fused nanobody could target more than one RBD antigen at the same time, and this multimeric nanobody-antigen structure could be more stable than the monomeric nanobody-antigen complex. This possibility has also been demonstrated by several recent studies of testing nanobody multimer against SARS-CoV-2 (51, 61). In addition, as a therapeutic candidate, our preliminary animal studies did not detect any systemic absorption of VHH-IgA1.1 in animal serum with nasal delivery (data not shown). Future work is now focused on assessing additional safety monitoring in pre-clinical toxicology studies.

The recent technological advances in large-scale manufacturing of nanobodies and their derivatives in plants and microorganisms, such as *P. pastoris*, position nanobody-based immunotherapy as a potentially efficient and cost-effective prophylactic for COVID-19 (62). Compared to conventional mammalian cell production, VHH-IgA can be produced in large quantities in yeast or soybean seed expression systems, and the efficacy of productions has been proven successful against mucosal *E. coli* infection in piglets (34). Due to the faster growth rate, high transformation efficiency and easy scalability, the yeast expression system would significantly reduce the time input and cost from nanobody identification and manufacturing to IND submission (63). Our study combines the methodology for rapid nanobody screening from display libraries and low-cost production systems to identify potent nanobodies against SARS-CoV-2, which could provide a more efficient and affordable platform that can be utilized to rapidly generate biologics against new emerging SARS-CoV-2 variants.

In this study, our results also demonstrated that VHH fused with the Fc region of human immunoglobulin, such as IgG and IgA, exhibited stronger binding activity and neutralization efficacy than the monomeric nanobody. Strikingly, IgA-Fc fusion offers significant benefits to the VHH compared to the IgG equivalent. This isotype related enhanced activity was also reported in our and other group’s previous studies (14, 40, 64, 65). VHH-IgG1.1 and VHH-IgA1.1 presented relatively identical ELISA binding EC_50_ in ELISA (**Figure S1E**), but the neutralization IC_50_ against those VOC was remarkably reduced (**Figure S1L**). One possibility could be the longer hinge region of IgA-Fc provides more flexibility to VHH to access certain type of epitopes (66), which are fully exposed in truncated RBD but buried in full-length trimeric spike protein. In this case, the benefits of IgA-Fc fragment enabled activity should be epitope specific and cannot be extrapolated to other antibodies. In this study, the underlying mechanism behind the increased potency of IgA-Fc fusion compared to IgG-Fc is still unclear, and this mechanism could be critical for nanobody design and development in the future, so future structural analysis of VHH1.1 against various RBD domain in both formats may be able to mechanistically evaluate these differences and indicate a fundamental rule for the nanobody drug development.

In summary, we have determined that antibody avidity and Fc domain can play a critical role in potency of nanobodies against SARS-CoV-2. Furthermore, our study provides the first proof of concept that nasal administration of a SARS-CoV-2 RBD targeting VHH-IgA confers broad protection against SARS-CoV-2 VOC. In addition, our preliminary animal studies did not detect any systemic absorption of VHH-IgA1.1 in animal serum with nasal delivery (data not shown). With ongoing pre-clinical safety studies, we will determine if VHH-IgA1.1 alone is safe for mucosal administration or need further humanization for systemic delivery. VHH-IgA has the flexibility to be produced in both mammalian and yeast systems for broader applications in future pandemics. Based on our results, we propose that mucosal (intranasal) delivery of VHH-IgA1.1 can serve as a non-invasive, safe, and cost-effective prophylactic or post-exposure treatment. Our ongoing pre-clinical and clinical translation of this platform will have important implications for protection against SARS-CoV-2 and other respiratory pathogens.

## Materials and methods

### S glycoprotein and VHH expression and purification

SARS-CoV-2 spike glycoproteins were expressed and purified as previously described (14). Briefly, the amino acid sequence of SARS-CoV-2 S glycoprotein (GeneBank: MN908947), VOC including D614G, Alpha (B.1.1.7) (69-70del, Y144del, N501Y, A570D, D614G, P681H, T716I, S982A, and D1118H), Beta (B.1.351) (L18F, D80A, D215G, 242-244del, R246I, K417N, E484K, N501Y, D614G and A701V), Gamma (P.1) (L18F, T20N, P26S, D138Y, R190S, K417T, E484K, N501Y, D614G, H655Y, T1027I, V1176F), Kappa (B.1.617.1) (E154K, L452R, E484Q, D614G, and P681R), Delta (B.1.617.2) (T192R, G142D, D156G, 157-158del L452R, T478K, D614G, P681R and D950N), Mu (B.1.621) (T95I, Y144S, Y145N, R346K, E484K, N501Y, D614G, P681H and D950N), Omicron (B.1.1.529) (A67V, 69-70del, T95I, G142D, 143-146del, 211del, L212I, 214insertEPE, G339D, S371L, S373P, S375F, K417N, N440K, G446S, S477N, T478K, E484A, Q493R, G496S, Q498R, N501Y, Y505H, T547K, D614G, H655Y, N679K, P681H, N764K, D796Y, N856K, Q954H, N969K and L981F) and human ACE2 (GeneBank: NM_001371415.1) sequence were used to design a codon-optimized version of the gene encoding the SARS-CoV-2 spike proteins as previously described (67, 68), including the full length spike protein (a.a. 1-1273), ectodomain trimer of spike protein (a.a.1-1208) and RBD domain (a.a. 319-541). The synthetic gene was cloned into pcDNA3.1 in-frame with Osteo signal peptide (MRAWIFFLLCLAGRALA) on the N-terminal and 6XHis tags on the C-terminal that enabled the purification. Nanobody sequences were cloned into pcDNA3.1 vector in-frame with Osteo leader on the N-terminal and 6XHis tags on C-terminal for monomeric nanobody or with human IgA-Fc and IgG-Fc for VHH-IgA and VHH-IgG fusion. All constructs were transient transfected into Expi293 cells using ExpiFectamine™ 293 Transfection Kit **(**ThermoFisher Scientific). The supernatant of transfected cell culture was harvested at day 4-5 post transfection, and protein in the supernatant were purified by immobilized metal chelate affinity chromatography using nickel-nitrilotriacetic acid (HisPur™ Ni-NTA Resin, ThermoFisher Scientific) agarose resin, the purified protein was eluted by 250mM imidazole in 1XPBS and was dialyzed in 1XPBS pH=7.2 overnight before the following assay.

### Elisa

Dilutions of purified antibody were tested in ELISA for reactivity against recombinant spike protein (14). Briefly, 96-well plates were coated with 100ul of S proteins at 5ug/ml in ELISA coating buffer (15mM Na₂CO₃ and 35mM NaHCO₃, pH=9.6) and was incubated overnight at 4°C. The plates were blocked with 1% BSA with 0.05% Tween-20 in PBS. Purified antibody diluted in blocking buffer (1× PBS plus 0.05% Tween 20 and 1% BSA) was added to the 96-well plates to incubate for 1 hour at room temperature. After 3X times washing with PBST (1XPBS with 0.05% Tween 20), plates were incubated with horseradish peroxidase-conjugated goat-anti-hIgA (1:30,000, Southern Biotech), horseradish peroxidase-conjugated goat-anti-hIgG (1:30,000, Southern Biotech) or anti-His (1:25000) for 1h at room temperature. The signal was developed using 100ul of 3, 3′, 5, 5′-tetramethylbenzidine reagents (Two components TMB, SeraCare). Absorbance at an optical density at 450 nm (OD450) was measured on an E_max_ precision plate reader (Molecular Devices) using Softmax software, and results were analyzed with GraphPad Prism 8.

### Non-reduced SDS-page

For denaturing non-reduced SDS-PAGE, 10μg of antibody samples were mixed with 2× sample loading buffer (Bio-Rad) and loaded on 12% Novex Tris-Glycine PAGE (Invitrogen). Electrophoresis was performed at room temperature for approximately 2h using a constant voltage (120 V) in running Tris-Glycine buffer until the dye front reached the end of the gel. The gels were stained using SYPRO™ Ruby (Invitrogen) and imaged in Chemi Doc XRS Imaging system (BioRad).

### Determination of antibody homogeneity by analytical size exclusion chromatography (SEC)

150μg of antibodies in 50μL of injection volume was chromatographed onto the TSKgel 3000SWxl SEC column (Tosoh Bioscience) on Alliance HPLC System (Waters). The flow rate was 1mL/min using SEC buffer (10mM Na_2_HPO_4_, 10mM KH_2_PO_4_, 140mM NaCl and 0.02% Sodium Azide at pH=6.85), and the protein absorbance at 280 nm was monitored for 16min. Molecular mass determination was calculated by reference to a protein standard for gel filtration (Bio-Rad).

### 3D structuring mapping of binding epitope

The 3-dimensional structure of the SARS-CoV-2 spike trimer in an open conformation with one receptor binding domain (RBD) exposed (PDB ID: 6VYB) (69) was used for mapping RBD mutations. For highlighting the binding interface of human ACE2 (hACE2) and RBD, 6VW1 (70) was used. ACE2 complex with SARS-CoV-2 spike trimer was achieved by superposing RBD domains in the two crystal structures, 6VYB and 6VW1. Figures were generated using PyMOL Molecular Visualization System v2.5.2 (Schrödinger).

### Bio-layer interferometry

The binding kinetics of antibodies to RBD were measured by BLI on an Octet HTX (PALL/ForteBio) as previously described (14). Antibodies were added to 96 wells plates at 1,000 nM and titrated 1:2 to 62 nM using PBS. RBD were biotinylated (EZ-Link™ Sulfo-NHS-LC-Biotinylation Kit, Thermo Fisher) and immobilized on Streptavidin (SA) Biosensors (ForteBio) for 120 seconds at 1,600 nM concentration. After a baseline step, antibody-antigen binding rate was determined when the biosensors with immobilized antigen were exposed to antibody at different concentrations for 120 seconds. Following association, the VHH-IgA1.1 and RBD complex was exposed to PBS and the rate of the antibody dissociation from the antigen was measured. Each assay was performed in triplicate. Binding affinities for VHH-IgA1.1 were calculated using association and dissociation rates with ForteBio Data analysis software v8.1 (PALL).

### hACE2 competition assays using biolayer interferometry

The protocol of hACE2 competition assays was adapted from the method described in (71). In brief, 10μg/ml biotinylated SARS-CoV-2 RBD was loaded on Streptavidin (SA) sensors (ForteBio) for 240 seconds. After 20s baseline measurement in 1X PBS, the sensors were dipped in 3-fold serially diluted antibodies starting from 5ug/ml for 200s. After that, the sensors were directly dipped in the purified hACE2 (a.a 1-615) solution (10μg/ml) for 200s to record the response signal. The blocking percentage at each concentration were calculated as: [1- normalized hACE2 response of antibody/normalized hACE2 response of IRR antibody] x100. The blocking IC_50_ values were calculated by nonlinear fit based on dose-blocking curves.

### Pseudotyped virus neutralization assay

Production of pseudotyped SARS-CoV-2 was performed as previously described (14, 72). Pseudovirus was generated by 2nd generation lentiviral packaging plasmids (psPAX2: Addgene Plasmid #12260 and pLenti-Luc: Addgene Plasmid #17477) which contain a luciferase gene to direct luciferase expression in target cells (73). Full length of SARS-CoV-2 spike protein (a.a.1-1273) were provided as envelop protein by co-transfection in 293T cells. Supernatant containing virus particles were harvested 48 h post-transfection and concentrated by Centricon^®^ Plus-70 Centrifugal Filter Units (EMD Millipore). Before assessing antibody neutralization, the 293T cells were transiently transfected with 100ng pcDNA-hACE2 full length each well in 96 well plates and incubated at 37°C for 24 h. On the following day, the titration of pseudovirus was performed on 293T cells expressing hACE2 receptor to determine the volume of virus needed to generate 1,000,000cps in the infection assay. To determine the neutralization activity of antibodies, the appropriate volume of pseudovirus was pre-incubated with serially diluted antibodies for 1 h at room temperature before being added to 293T cells expressing hACE2. 48 hours post virus induction, the infection was quantified by luciferase detection with Bright Glo luciferase assay (Promega) and read in a Victor3 plate reader (Perkin Elmer) for light production.

### Plaque reduction neutralization test (PRNT)

Wadsworth Center PRNTs were conducted as previous described (74) in the BSL-3 lab by mixing 100ul of approximately 120-180 plaque forming units of SARS-CoV-2, isolate USA-WA1/2020 (BEI Resources, NR181 52281), low Lineage D614G patient isolate (hCoV-19/USA/NY-Wadsworth-20005877-01/2020), low passage Lineage Alpha (B.1.1.7) patient isolate (hCoV-19/USA/NY-Wadsworth-20291673-01/2020), low passage Lineage Omicron (BA.1) patient isolate (hCoV-19/USA/NY-Wadsworth- 21103366-01/2021) with 100 ul of 2-fold serially diluted test sera and incubated at 37°C in 5% CO2 for one hour. Confluent Vero E6 cells (C1008, ATCC CRL-1586) or Vero E6 with TMPRSS2 cells (JCRB1819, Sekusi XeonTech) seeded in 6 well plates were inoculated with 100ul of the virus:serum mixture and adsorption proceeded for one hour at 37°C in 5% CO2. A 0.6% agar overlay prepared in maintenance medium (Eagle’s Minimal Essential Medium or Dulbecco’s Modified Eagle Medium, 2% heat-inactivated fetal bovine serum, 100 µg/ml Penicillin G, 100 U/ml Streptomycin, 1mg/ml Geneticin) was added after adsorption and the assay was incubated at 37°C in 5% CO2. A second agar overlay with 0.2% Neutral red added was added two days post infection. After an additional day of incubation, the number of plaques in each well were recorded. The discrete titer was reported as the inverse of the highest dilutions of sera providing 50% (PRNT50) or 90% (PRNT90) viral plaque reduction relative to virus-only infection. IC_50_ values were calculated by determining the percent neutralization (relative to virus only controls) for technical duplicates of each well and using non-linear regression to calculate the IC_50_ ([inhibitor] vs. normalized response with variable slope, Graphpad Prism). Three or more independent biological replicates with technical duplicates were completed for each antibody. Normal human serum was used as a negative control and previously characterized COVID-19 patient sera or monoclonal antibody was used as a positive control in each assay.

### Biosafety

All study protocols were reviewed and approved by the Environmental Health and Safety and Institutional review board at University of Massachusetts Chan Medical School prior to study initiation. All experiments with SARS-CoV-2 were performed in a biosafety level 3 laboratory by personnel equipped with powered air-purifying respirators.

### Mice

All animal experiments were approved by the Institutional Animal Care Use Committees at the University of Massachusetts Chan Medical School. Animals were kept in a specific pathogen free (SPF) environment. Hemizygous K18-hACE2 C57BL/6J mice (strain: 2B6.Cg-Tg (K18-ACE2)2Prlmn/J) were obtained from The Jackson Laboratory (75–77). Animals were housed in groups and fed standard chow diets. Sample sizes used are in line with other similar published studies (78).

### SARS-CoV-2 infection and antibody protection

T75 flaks of VeroE6 cells were infected with the USA-WA1/2020 (NR-52281; BEI Resources), hCoV-19/USA/MD-HP20874/2021 (Lineage B.1.1.529; Omicron Variant, NR-56461; BEI Resources) at an MOI of 0.01 for 48 hours. Supernatants were centrifuged at 1500g for 10 minutes and aliquoted and stored at -80°C. Virus titre was determined by TCID50 assay in VeroE6 cells. B.1.1.7 (Alpha variant) strain was kindly provided by Kristen St. George, Department of health, Wadsworth Center. For the animal protection, 8–12-week-old male and female mice, were anaesthetized with intraperitoneal injection of ketamine (100 mg kg^−1^ body weight) and xylazine (10 mg kg^−1^ body weight). Mice were then infected intranasally with 2.5 × 10^4^ PFU of SARS-CoV-2. Nanobodies were intranasally delivered at 1h prior; or 6 or 12h post viral infection. Mice were monitored daily for weight loss and survival for 10 days after challenge.

### SARS-CoV-2 RNA analysis

For viral load analysis, a separate group of mice were euthanized two days post infection in isoflurane. Lung tissue was placed in a bead homogenizer tube with 1 ml of MEM + 2% FBS. After homogenization, 100 μl of this mixture was placed in 300 μl Trizol LS (Invitrogen) and RNA was extracted with the direct-zol RNA miniprep kit (Zymo) per the manufacturer’s instructions. Quantification of SARS-CoV-2 RNA levels was performed using the iScript cDNA synthesis kit (Bio Rad). 5ng of cDNA was then subjected to qPCR analysis using iQ SYBR Green super-mix reagent (Bio Rad). Gene expression levels were normalized to TATA-binding protein (TBP) or HPRT. Relative mRNA expression was calculated by a change in cycling threshold method as 2^-ddC (t)^. Specificity of RT-qPCR amplification was assessed by melting curve analysis. The sequences of primers used in this study are as follows. SARS-Cov-2-N F CTCTTGTAGATCTGTTCTCTAAACGAAC, R GGTCCACCAAACGTAATGCG. Nsp14 F TGGGGYTTTACRGGTAACCT, R AACRCGCTTAACAAAGCACTC. ORF1 F GAGAGCCTTGTCCCTGGTTT, R AGTCTCCAAAGCCACGTACG.

### Screening and establishment of *P. pastoris* cell line to express VHH-IgA1.1

VHH-IgA1.1 was expressed in *P. pastoris* using the PichiaPink expression system (Life Technologies) following the manufacturer’s protocol. Briefly, antibody genes were codon-optimized for expression in *P. pastorius* and VHH and IgA (excluding the CH1 region) were inserted in frame with the *S. cerevisiae* α-mating factor pre-sequence secretion signal downstream of the *AOX1* promoter in the pPINKα-HC vector. Plasmid DNA linearized with *Spe*I was used to electroporate stable yeast clones, and expression of the antibody gene was determined by *ADE2* complementation on PAD selection plates. For small scale selection of high expressing clones, we utilized a high throughput approach (79). Colonies expressing the antibody gene were picked from selection PAD plates and grown in 1mL YPD media in deep well plates (square, U-bottom) at 30^0^C on shaker plates (1000 rpm, 3mm throw) overnight. Plates were covered with a breathable membrane to permit adequate aeration at all steps. 50µL overnight culture was transferred to new plates with BMGY media and grown for 48 hours at 25^0^C as above. Following 48 hour incubation BMGY plates were pelleted and cultures resuspended in new deep well plates contain BMMY media for 96 hours at 25^0^C as above. Production of antibody was tested every 24 hours to determine optimal harvest time. All time points were tested for expression of VHH-IgA1.1 by RBD ELISA using Expi293 cells produced VHH-IgA1.1 as a standard.

### Large scale antibody production and purification from *P. pastoris*

Large scale expression was carried out according to the manufacturer’s protocol. In brief, selected clones were grown overnight in BMGY at 30^0^C before scaling up to 1L BMGY at 25^0^C on shaker flasks (250 rpm) and grown to an OD_600_ of 4-10.0. To induce, cells were pelleted and resuspended in BMMY at 25^0^C in two baffled 1L flasks (100mL each) for 48 hours, with 100% methanol added to 0.5% every 24 hours. Following induction cultures were spun down and supernatants harvested, filtered, and stored at -80^0^C until purification. VHH-IgA1.1 in the supernatant was purified by immobilized affinity chromatography using Peptide M/Agarose beads and dialyzed in PBS pH=7.2.

### Statistical analysis

Statistical calculations were performed using Prism version 8.1.1 (GraphPad Software, La Jolla, CA). EC_50_ and IC_50_ values were calculated by sigmoidal curve fitting using nonlinear regression analysis. For comparisons of two groups, two-tailed student’s tests were performed. Multiple comparison analysis was performed using two-way ANOVA. 3 to 16 mice were used per experiment, sufficient to calculate statistical significance, and in line with similar studies published in the literature. Randomization and blinding was not performed.

## Data availability statement

The original contributions presented in the study are included in the article/**Supplementary Material.** Further inquiries can be directed to the corresponding authors.

## Ethics statement

The animal study was reviewed and approved by IACUC committed at UMass Chan Medical School.

## Author contributions

Conceptualization: YW, KF, LC, MK, and FH; Formal Analysis: QL, FH, RG, AW, ME, and AA; Investigation: QL, FH, RG, AW, ME, AA, CM, ZM, JZ, NY, AM, AD, AP, and KM; Methodology: QL, FH, RG, AW, CT, AK, BP, KF, JC, AD, AP, KM, and YW; Project administration: ZS, YW, and LC; Supervision: YW, KF, LC, and MK; Visualization: QL, FH, and YW; Writing – original draft: QL, FH, AA, ZS, YW, KF, LC, and MK; Writing – review and editing: QL, FH, YW, KF, LC, MK, and ZS. All authors contributed to the article and approved the submitted version.

## Acknowledgments

Research reported in this publication was supported by NIAID of the National Institutes of Health under award number R01AI159182. This work was also supported by funding from the Massachusetts Consortium on Pathogen Readiness (MassCPR) and pilot funds from the UMass Chan Pandemic Research Fund. The *in vitro* PRNT experiments were supported by NIH 1U01CA260508-01 and CDC NU50CK000516. We also thank Dr. Kirsten St. George from Department of Health, Wadsworth Center for kindly providing the SARS-CoV-2 Alpha variant.

## Conflict of interest

The authors declare that the research was conducted in the absence of any commercial or financial relationships that could be construed as a potential conflict of interest.

## Publisher’s note

All claims expressed in this article are solely those of the authors and do not necessarily represent those of their affiliated organizations, or those of the publisher, the editors and the reviewers. Any product that may be evaluated in this article, or claim that may be made by its manufacturer, is not guaranteed or endorsed by the publisher.

## Author disclaimer

The content is solely the responsibility of the authors and does not necessarily represent the official views of the National Institutes of Health.
