# Supplementary material for: Mucosal nanobody IgA as inhalable and affordable prophylactic and therapeutic treatment against SARS-CoV-2 and emerging variants
Source: Front Immunol. 2022 Sep 12;13:995412. doi: 10.3389/fimmu.2022.995412 (PMC9512078; doi:10.3389/fimmu.2022.995412)
Supplement: Supplementary file 1 [file DataSheet_1.docx]

**Supplemental Figures**

**
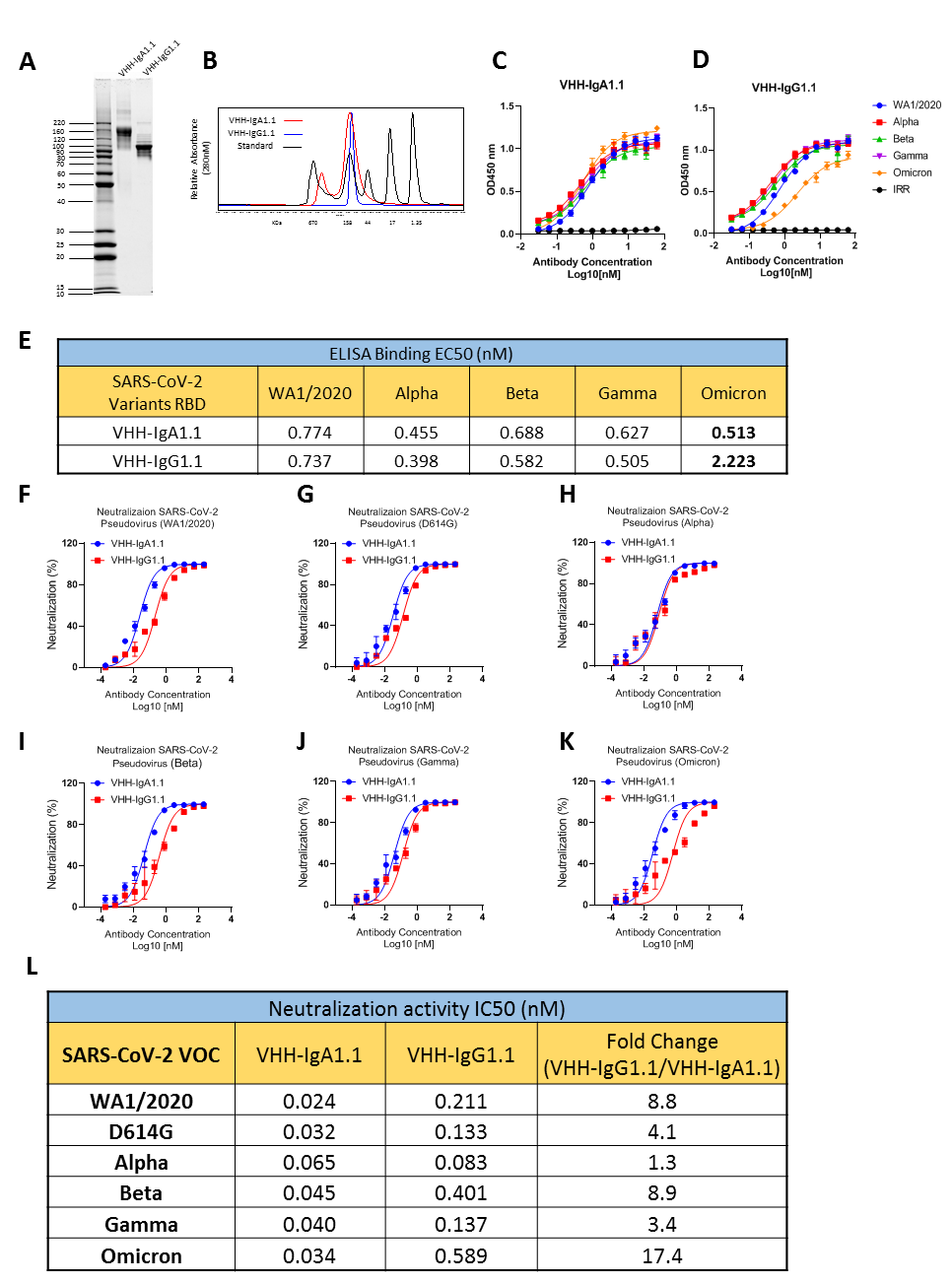
**

**Fig.S1 Comparison of VHH-IgG1.1 and VHH-IgA1.1 against SARS-CoV-2 VOC.**

The purity and homogeneity of purified VHH-IgA1.1 and VHH-IgG1.1 were evaluated by non-reduced SDS-PAGE **(A)** and HPLC-SEC **(B)**. **(C-D)** Comparison of VHH-IgA1.1 and VHH-IgG1.1 binding with RBD domain of SARS-CoV-2 VOC in ELISA. **(F-K)** Head to head comparison of VHH-IgA1.1 and VHH-IgG1.1 against pseudovirus of indicated SARS-CoV-2 VOC. Pseudoviral transduction was measured by luciferase activities to calculate neutralization (%) relative to non-antibody-treated controls. **(E and L)** Data was plotted as the average ± SD, and IC_50_ values were calculated by nonlinear regression analysis. Three or more independent biological replicates were completed for each antibody.


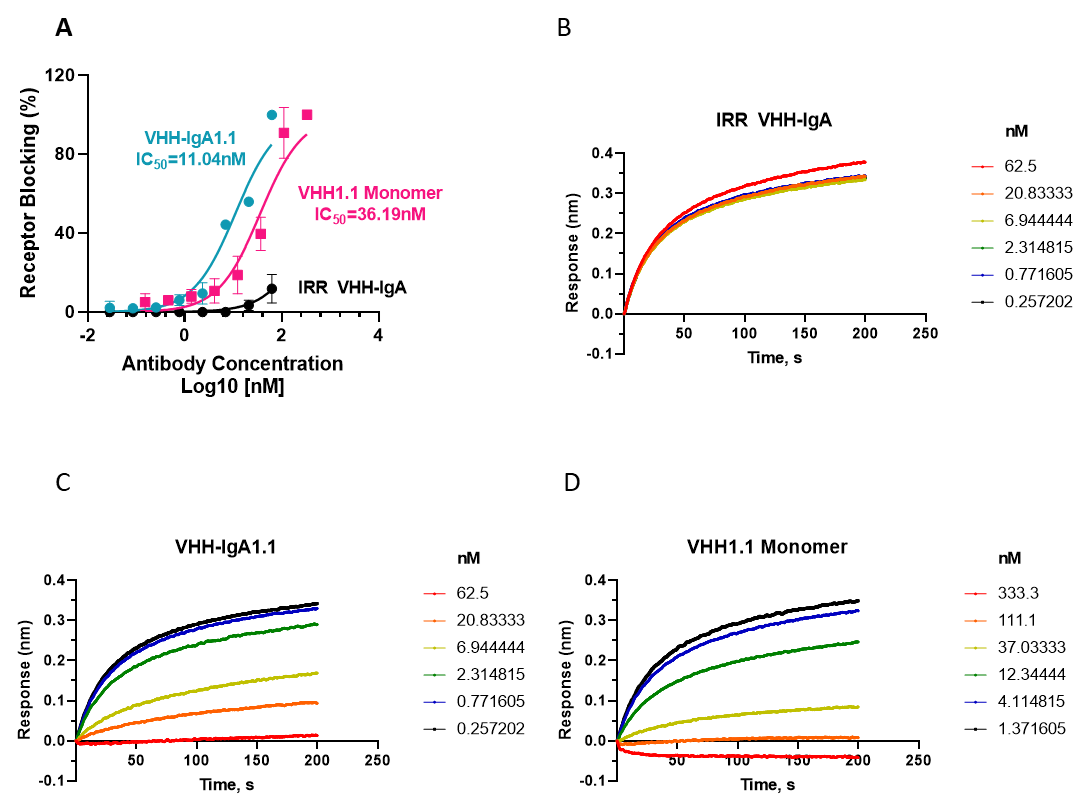


**Fig. S2 VHH-IgA1.1 block the interaction of SARS-CoV-2 RBD and hACE2 receptor.**

**(A)** Bio-layer interferometry (BLI) traces measuring hACE2 competition for SARS-CoV-2 RBD binding in the presence of indicated antibodies. Representative normalized hACE2 response curves blocked by negative control IRR VHH-IgA **(B),** VHH-IgA1.1 **(C)** and VHH1.1 Monomer **(D).**


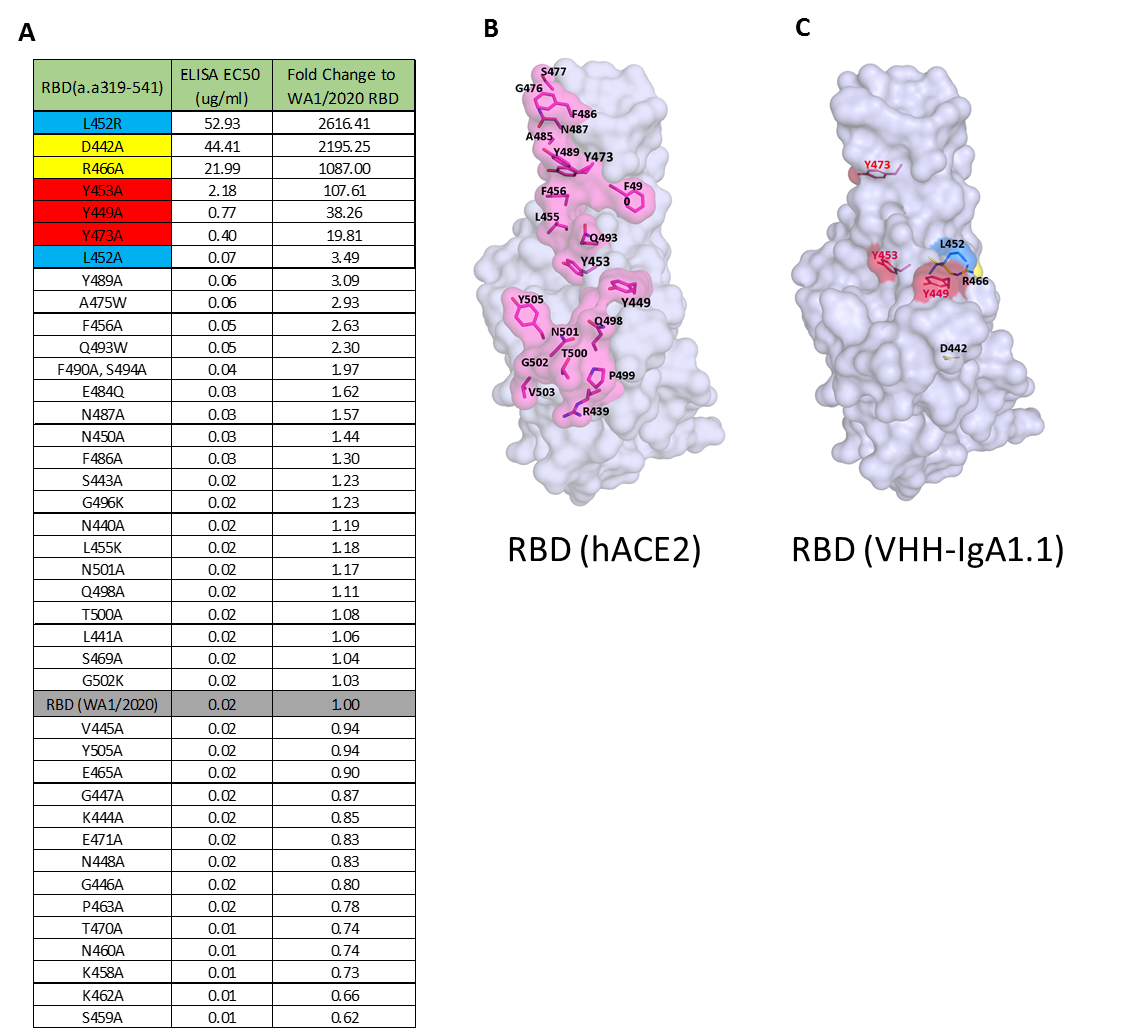


**Fig. S3 Mutational guided molecular modeling of VHH-IgA1.1 binding to RBD.**

**(A)** RBD carrying single residue mutations was expressed as recombinant proteins. ELISAs assay were performed to determine the effect of the mutated residues on RBD binding to VHH-IgA1.1. Key residues (Red, Blue and Yellow) were identified as RBD mutations that reduced EC50 values to the RBD (Gray) of WA1/2020 strain. EC50 values calculated from three independent experiments. **(B)** The binding interface on SARS-CoV-2 RBD with hACE2, the key residues are labeled with red color. **(C)** Key residues of VHH-IgA1.1 binding with SARS-CoV-2 RBD identified by ELISA are marked by color according to influence. Red represents overlap with hACE2 interface, blue suggests residues known to disrupt ACE2 binding indirectly, yellow are residues influence the bind of VHH-IgA1.1 with RBD but are out of hACE2 interface.

**
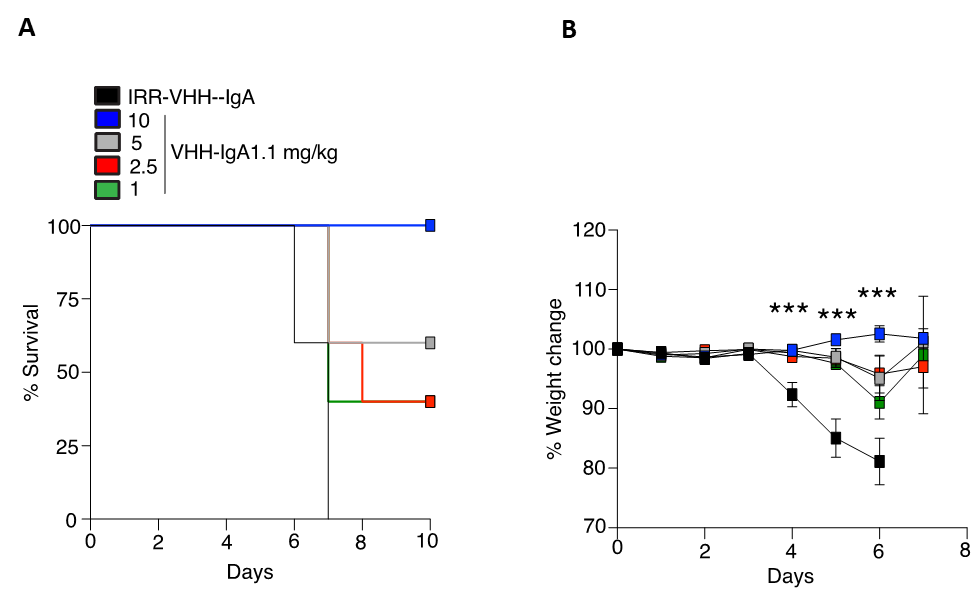
**

**Fig. S4** **Dose range evaluation of VHH-IgA1.1 in K18-ACE2 SARS-CoV-2 infection model.**

Survival **(A)** and weight loss **(B)** of K18-ACE2 transgenic mice infected intranasally with SARS-CoV-2 (2.5x10^4^ PFU/mouse) with a 1 h intranasal pre-treatment of 10mg/kg IRR-IgA1 isotype control (*n=5*), or VHH-IgA1.1 (*n=5*) at the indicated doses. ***, *P*<0.0001, Error bars show means ± SEM.


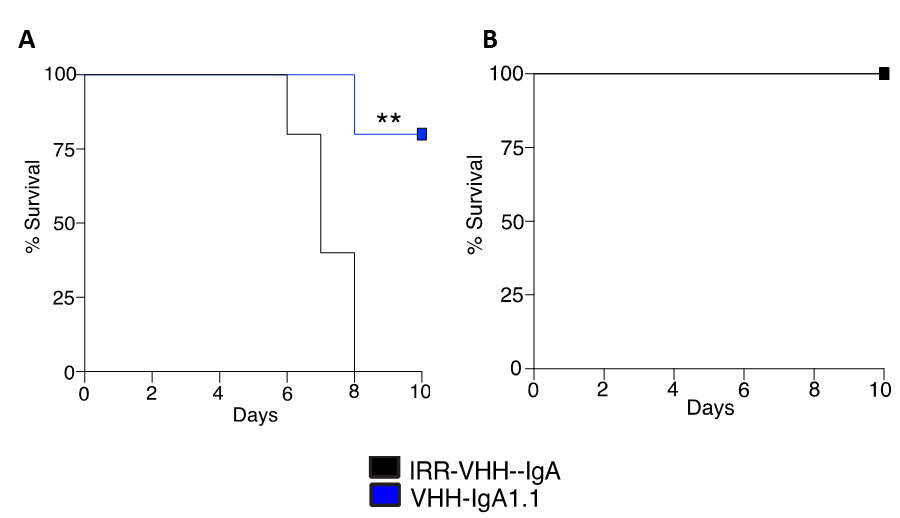


**Fig. S5 Survival cure of VHH-IgA1.1 protected transgenic mice from SARS-CoV-2 infection**

Survival of K18-ACE2^+/-^ transgenic mice infected intranasally with Alpha (B.1.1.7) (2.5x10^4^ PFU/mouse) **(A)** and Omicron (B.1.1.529) (1x10^5^ PFU/mouse) **(B)** variant of SARS-CoV-2 followed by pre-treatment of 10mg/kg IRR-IgA1 isotype control (*n=5*) or VHH-IgA1.1 (*n=5*).
